# Identification and Characterization of MicroRNAs in Snakehead Fish Cell Line upon Snakehead Fish Vesiculovirus Infection

**DOI:** 10.3390/ijms17020154

**Published:** 2016-01-26

**Authors:** Xiaodan Liu, Jiagang Tu, Junfa Yuan, Xueqin Liu, Lijuan Zhao, Farman Ullah Dawar, Muhammad Nasir Khan Khattak, Abeer M. Hegazy, Nan Chen, Vikram N. Vakharia, Li Lin

**Affiliations:** 1 Department of Aquatic Animal Medicine, College of Fisheries, Huazhong Agricultural University, Wuhan 430070, China; l910110526@126.com (X.L.); jfyuan@mail.hzau.edu.cn (J.Y.); xueqinliu@mail.hzau.edu.cn (X.L.); zhaolijuan4234@163.com (L.Z.); farmandawar2012@yahoo.com (F.U.D.); abeer@nwrc-egypt.org (A.M.H.); chennan@mail.hzau.edu.cn (N.C.); 2 Freshwater Aquaculture Collaborative Innovation Center of Hubei Province, Wuhan 430070, China; 3 Department of Zoology, Hazara University, Mansehra, Khyber Pakhtoonkhwa 21300, Pakistan; mnasir43663@googlemail.com; 4 Central Laboratory for Environmental Quality Monitoring (CLEQM), National Water Research Center (NWRC), Cairo 13621, Egypt; 5 Institute of Marine and Environmental Technology, University of Maryland, Baltimore, MD 21202, USA; vakharia@umbc.edu

**Keywords:** snakehead fish Vesiculovirus, SSN-1 cell, miRNA, deep sequencing

## Abstract

MicroRNAs (miRNAs) play important roles in mediating multiple biological processes in eukaryotes and are being increasingly studied to evaluate their roles associated with cellular changes following viral infection. Snakehead fish Vesiculovirus (SHVV) has caused mass mortality in snakehead fish during the past few years. To identify specific miRNAs involved in SHVV infection, we performed microRNA deep sequencing on a snakehead fish cell line (SSN-1) with or without SHVV infection. A total of 205 known miRNAs were identified when they were aligned with the known zebrafish miRNAs, and nine novel miRNAs were identified using MiRDeep2 software. Eighteen and 143 of the 205 known miRNAs were differentially expressed at three and 24 h post-infection (poi), respectively. From the differentially-expressed miRNAs, five were randomly selected to validate their expression profiles using quantitative reverse transcription polymerase chain reaction (qRT-PCR), and their expression profiles were consistent with the microRNA sequencing results. In addition, the target gene prediction of the SHVV genome was performed for the differentially-expressed host miRNAs, and a total of 10 and 58 differentially-expressed miRNAs were predicted to bind to the SHVV genome at three and 24 h poi, respectively. The effects of three selected miRNAs (miR-130-5p, miR-214 and miR-216b) on SHVV multiplication were evaluated using their mimics and inhibitors via qRT-PCR and Western blotting. The results showed that all three miRNAs were able to inhibit the multiplication of SHVV; whereas the mechanisms underlying the SHVV multiplication inhibited by the specific miRNAs need to be further characterized in the future.

## 1. Introduction

miRNAs, which are extensively expressed in various organisms, comprise a class of endogenous non-coding RNAs and post-transcriptionally regulate gene expression via translational repression and/or degradation of mRNAs by binding to complementary sequences in the 3′ untranslated region (UTR) of mRNAs [1,2,3,4,5,6]. MiRNAs are initially transcribed as long pri-miRNAs in the nucleus and then processed by the RNase III enzyme Drosha in the nucleus and Dicer in cytoplasm to be mature ~22 nt miRNAs [7]. To date, >10,000 miRNAs have been annotated in 96 species [8], including >1000 human miRNAs [9]. It is believed that >50% of host mRNAs are regulated by one or more miRNAs, and individual miRNAs regulate >100 mRNAs [5]. miRNAs are involved in almost all biological processes in eukaryotes, including cell differentiation, apoptosis and immunity [10,11,12]. In addition, miRNAs are also involved in the modulation of gene expression and replication of viruses and play a pivotal role in host-virus interactions [1,13]. Therefore, the study of miRNA-mediated host-virus interactions is important to understand the mechanisms of virus infection and host counteraction [14].

Members of the family Rhabdoviridae, such as spring viremia of carp virus, infectious hematopoietic necrosis virus, viral hemorrhagic septicemia virus and Siniperca chuatsi rhabdovirus, have caused serious diseases in a wide variety of species in aquaculture [15,16,17]. In 2014, a disease outbreak occurred in hybrid snakehead fish cultured at a farm in Guangdong province, China. From the diseased snakehead fishes, a virus was isolated and determined to be Vesiculovirus via genome sequencing. The genome of snakehead Vesiculovirus (SHVV) is about 11 kb in length and encodes five structural proteins: nucleoprotein (N), phosphoprotein (P), matrix protein (M), glycoprotein (G) and RNA-dependent RNA polymerase protein (large protein, L) [18,19]. In addition to the genome sequence, a snakehead fish cell line SSN-1, but not a zebrafish cell line (ZF4), was able to support efficient replication of SHVV [18,20]. However, the pathogenic mechanism of SHVV infection is still poorly understood. Therefore, we aimed to identify the miRNA expression profile of SSN-1 cells upon SHVV infection in this study. In addition, several miRNAs of interest were chosen, and their roles in SHVV infection were further investigated.

## 2. Results

### 2.1. Overview of the Illumine Hiseq2500 Sequencing Data of Small RNAs

**Figure 1:**
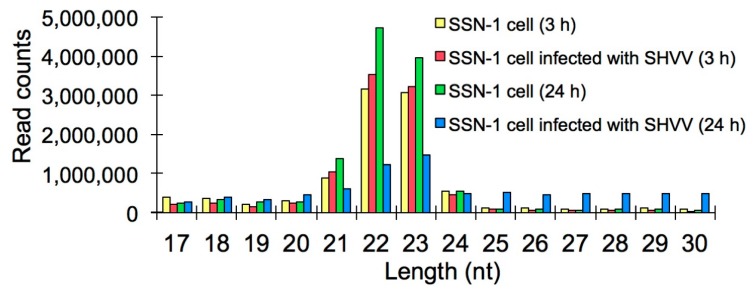
Length distribution of small RNAs derived from SSN-1 cells with or without snakehead fish Vesiculovirus (SHVV) infection. The small RNAs from the indicated four samples with lengths between 17 and 30 nt are analyzed.

To investigate the miRNA expression profiles of snakehead fish cell line SSN-1 with or without SHVV infection, small RNA libraries from SHVV infected or non-infected SSN-1 cells at three and 24 h post-infection (poi) were sequenced by illumine Hiseq2500. About 10 million clean reads, which were passed through quality filtering, adapter filtering and length filtering, were obtained from each of the four samples, including SSN-1 cells (3 h), SSN-1 cells infected with SHVV (3 h), SSN-1 cells (24 h) and SSN-1 cells infected with SHVV (24 h) (Table S1). Given that no genome of snakehead fish was available, the unique clean reads were subjected to the alignment with the genome of zebrafish. The results showed that the aligned reads, which can be mapped to the genome of zebrafish, account for only 20%–40% of the total unique clean reads (Table S1). The length distribution of the clean reads was analyzed, and a similar trend of the length distribution was observed in the four samples. Most of the small RNAs were 21–23 nt in length (Figure 1). 

### 2.2. Identification of Conserved miRNAs in SSN-1 Cells

**Figure 2:**
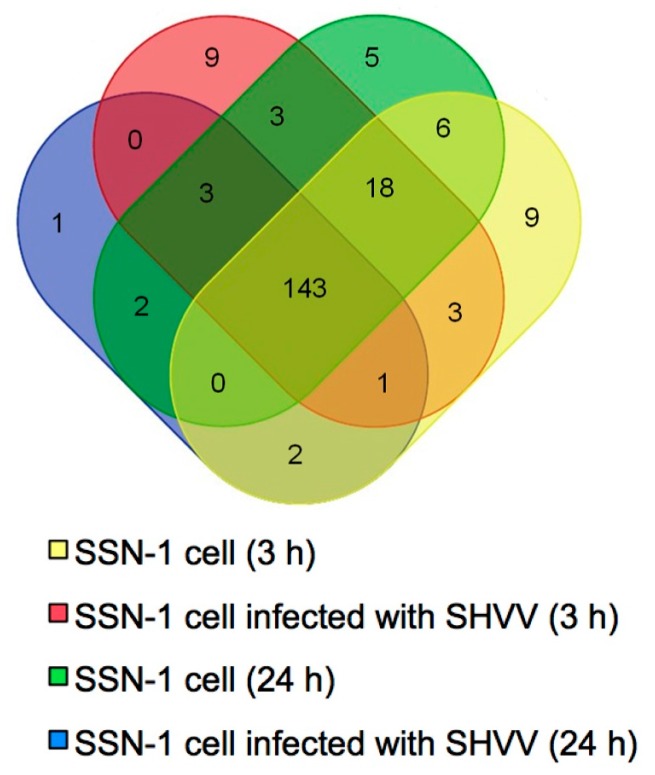
Identification of miRNAs compared to zebrafish miRNAs. miRNAs from the four samples were compared to the known zebrafish miRNAs in miRBase 20.0. A total of 205 known miRNAs were clustered including 182 miRNAs from sample SSN-1 cells (3 h), 180 miRNAs from sample SSN-1 cells infected with SHVV (3 h), 180 miRNAs from sample SSN-1 cells (24 h) and 152 miRNAs from sample SSN-1 cells infected with SHVV (24 h). The Venn diagram shows the distribution of miRNAs of the four samples. The overlapping section represents the number of co-expressed miRNAs.

To identify conserved miRNAs in SSN-1 cells, the data from the four samples were aligned with the known zebrafish miRNAs in miRBase 20.0. A total of 205 miRNAs belonging to 102 families were identified (Excel S1). There were 182 miRNAs from sample SSN-1 cells (3 h), 180 miRNAs from sample SSN-1 cells infected with SHVV (3 h), 180 miRNAs from sample SSN-1 cells (24 h) and 152 miRNAs from sample SSN-1 cells infected with SHVV (24 h) (Figure 2). Among the 205 conserved miRNAs, 143 miRNAs were present in all four samples (Figure 2). miRNAs let-7a, miR-100-5p, miR-10b-5p, miR-125b-5p, miR-146a, miR-181a-5p, miR-21, miR-27c-3p and miR-92a-3p were the most abundant miRNAs (>100,000 reads) in the four samples (Excel S1). However, the expression levels of miR-124-3p, miR-124-5p, miR-124-6-5p, miR-133a-3p, miR-133b-3p, miR-135b-3p, miR-135b-5p, miR-137-3p and some other miRNAs were rather low (Excel S1). These results indicated that the miRNAs exhibited a wide range of expression levels.

### 2.3. Prediction and Validation of Novel miRNAs

**Figure 3:**
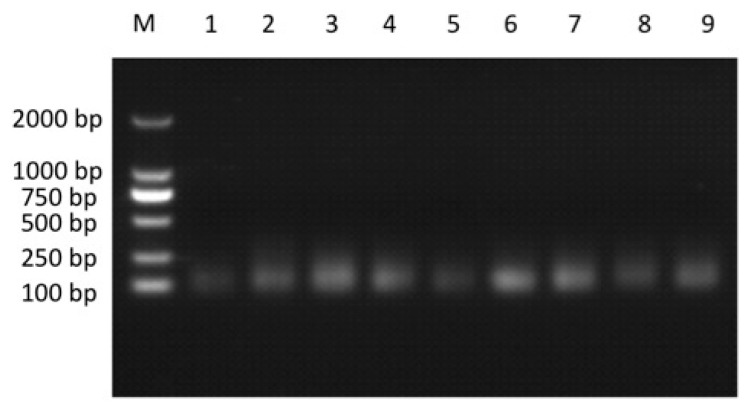
Validation of nine novel miRNAs by RT-PCR. M: marker; Lanes 1–9: the RT-PCR products of the nine novel miRNAs.

After eliminating the known miRNAs, tRNA, snRNA, rRNA, cRNA, the non-annotated small RNAs that could be mapped to the genome of zebrafish were subjected to novel miRNA prediction using MiRDeep2 software. Nine novel miRNAs were identified (Table 1). In order to validate the existence of the nine novel miRNAs, RT-PCR was used, and the size of RT-PCR products was around 100 bp (Figure 3), indicating the existence of these novel miRNAs in SSN-1 cells.

**Table 1:** Summary of the predicted novel miRNAs.

	Sequence (5′→3′)	Mature Reads	Fold Change (Log_2_^I24/C24^) *	*p*-Value
1	UCCAUCAGUCACGUGACCUAC	26	−2.15182	2.03 × 10^−16^
2	UCGGGUCGCUAAUGACGUCACC	30	−3.57979	4.64 × 10^−21^
3	ACCAGGUGCUGUAAGCUU	67	<1	–
4	CUUUUAAUCUGAGGGUCCA	12	<1	–
5	AUGACUCGAACCCGAGGACUCG	13	1.57979	0.019847
6	AUCCGGCUCGAAGGACCAA	157	−4.36217	0
7	AAACACUGCCAGCUGCCACA	5	−3.67933	0.00165
8	GGGGCCUGAGUCCUUCUG	17	13.75742	0
9	ACCCCACUCCUGGUACCA	51	−4.59089	8.16 × 10^−197^

* I24: SSN-1 cell infected with SHVV (24 h); C24: SSN-1 cells (24 h).

### 2.4. Differentially-Expressed miRNAs and Validation of the miRNAs by qRT-PCR

**Figure 4:**
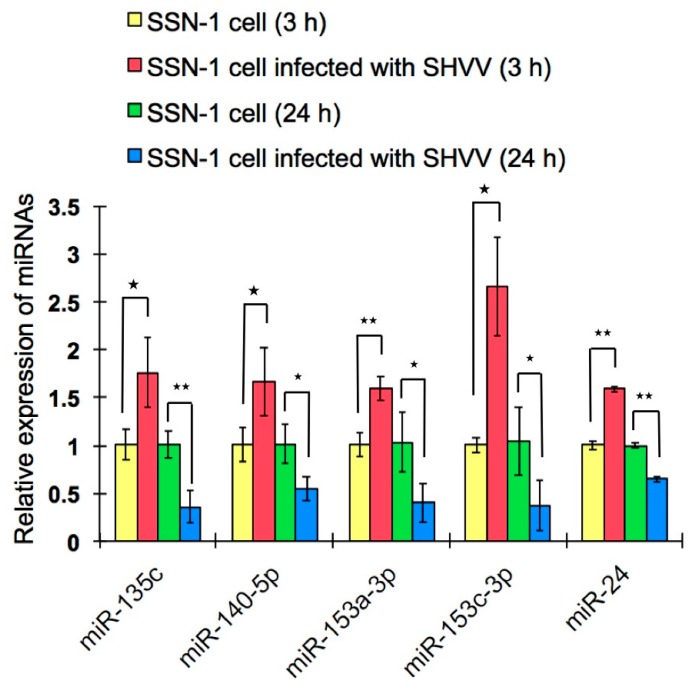
Expression analysis of five selected miRNAs by qRT-PCR. Five differentially-expressed miRNAs were randomly selected to quantify their expression profiles using qRT-PCR. Non-infected SSN-1 cells were used as the control. * and ** respectively indicate statistically-significant differences between infected and non-infected samples (* *p* < 0.05; ** *p* < 0.01).

The differentially-expressed miRNAs, including known and novel miRNAs between SHVV infected and non-infected SSN-1 cells, were analyzed. At three hours poi, 18 known miRNAs were differentially expressed, including 15 upregulated and three downregulated miRNAs (Table S2). No novel miRNA was differentially expressed at three hours poi. At 24 h poi, 150 miRNAs, including seven novel and 143 known miRNAs, were differentially expressed (Table 1 and Table S3). Of the seven novel differentially-expressed miRNAs, five were downregulated, and two were upregulated; whereas all of the 143 known differentially-expressed miRNAs were downregulated (Table S3). In order to validate the results of deep sequencing, five miRNAs, which were differentially expressed both at three and 24 h poi, were selected to quantify their expression via qRT-PCR. The results showed that the expression profiles of these five miRNAs were consistent with those obtained by deep sequencing (Figure 4).

### 2.5. miRNA Targets Prediction

To better understand the functions of miRNAs of snakehead fish (*Channa striatus*), especially their roles in SHVV multiplication, the targets of miRNA on the SHVV genome were predicted using Miranda software. At three hours poi, 10 of the 18 differentially-expressed miRNAs were predicted to bind to the SHVV genome. Most of them bind to one viral gene, except for miR-199-5p and miR-153a-3p, which were predicted to bind to two viral genes (Table S4). For the 143 differentially-expressed miRNAs at 24 h poi, 58 miRNAs were predicted to bind to viral genes. Twelve of them were predicted to bind to at least two genes of SHVV (Table 2). These data provided valuable information for further study on whether and how these miRNAs affect SHVV multiplication.

**Table 2:** Summary of targeted virus gene prediction of the differentially-expressed miRNAs when the sample SSN-1 cells infected with SHVV (24 h) were compared to SSN-1 cells (24 h).

Name	Sequence (5′→3′)	Target	Fold Change (Log_2_^I24/C24^) *
miR-23a-3-5p	GGAUUCCUGGCAGAGUGAUUU	N, L	−7.92953
miR-199-5p	CCCAGUGUUCAGACUACCUGUUC	N, L	−3.96545
miR-338	UCCAGCAUCAGUGAUUUUGUUG	N, L	−4.16476
miR-145-3p	GGAUUCCUGGAAAUACUGUUCU	N, L	−6.05784
miR-100-3p	CAAGCUUGUAUCUAUAGGUAUC	N, G	−3.61793
miR-216b	UAAUCUCUGCAGGCAACUGUGA	N, G, L	−6.73689
miR-130c-5p	GCCCUUUUUCUGUUGUACUACU	N, G, L	−4.79246
miR-214	ACAGCAGGCACAGACAGGCAG	N, P	−4.27387
miR-731	AAUGACACGUUUUCUCCCGGAUCG	N	−3.18425
miR-29b	UAGCACCAUUUGAAAUCAGUGU	P	−3.20471
miR-29a	UAGCACCAUUUGAAAUCGGUUA	P	−3.25812
miR-135c	UAUGGCUUUCUAUUCCUAUGUG	M	−1.6469
miR-145-5p	GUCCAGUUUUCCCAGGAAUCCC	G	−7.017
miR-92b-3p	UAUUGCACUCGUCCCGGCCUCC	G	−4.75081
miR-153b-3p	UUGCAUAGUCACAAAAAUGAGC	G, L	−2.41897
miR-7147	UGUACCAUGCUGGUAGCCAGU	G	−4.7948
miR-92a-3p	UAUUGCACUUGUCCCGGCCUGU	G	−3.97976
miR-184	UGGACGGAGAACUGAUAAGGGC	G	−2.16476
miR-301c-3p	CAGUGCAAUAGUAUUGUCAUAG	G	−2.45693
miR-363-3p	AAUUGCACGGUAUCCAUCUGUA	G	−3.26429
miR-454b	UAGUGCAAUAUUGCUUAUAGGG	G	−2.98676
miR-301a	CAGUGCAAUAGUAUUGUCAAAG	G	−2.1425
miR-130a	CAGUGCAAUGUUAAAAGGGCAU	G	−3.11229
miR-23a-5p	GAAUUCCUGGCAGAGUGAUUU	G, L	−6.09946
miR-153a-3p	UUGCAUAGUCACAAAAGUGAUC	G, L	−2.95278
miR-25-3p	CAUUGCACUUGUCUCGGUCUGA	G	−2.31239
miR-730	UCCUCAUUGUGCAUGCUGUGUGU	G	−4.60532
miR-204-5p	UUCCCUUUGUCAUCCUAUGCCU	G, L	−3.14817
miR-301b-3p	CAGUGCAAUAGUAUUGUCAUUG	G	−4.92954
miR-724	UUAAAGGGAAUUUGCGACUGUU	L	−2.63081
miR-181c-5p	CACAUUCAUUGCUGUCGGUGGG	L	−1.84696
miR-125a	UCCCUGAGACCCUUAACCUGUG	L	−3.16476
miR-183-5p	UAUGGCACUGGUAGAAUUCACUG	L	−1.57979
miR-199-3p	UACAGUAGUCUGCACAUUGGUU	L	−2.97786
miR-26a-2-3p	CCUAUUCAUGAUUACUUGCACU	L	−3.8599
miR-140-3p	UACCACAGGGUAGAACCACGGAC	L	−3.03951
miR-146a	UGAGAACUGAAUUCCAUAGAUGG	L	−1.98658
miR-150	UCUCCCAAUCCUUGUACCAGUG	L	−2.63751
miR-221-3p	AGCUACAUUGUCUGCUGGGUUUC	L	−2.48955
miR-34a	UGGCAGUGUCUUAGCUGGUUGU	L	−2.5756
miR-101b	UACAGUACUAUGAUAACUGAAG	L	−2.73747
miR-9-5p	UCUUUGGUUAUCUAGCUGUAUGA	L	−1.54182
miR-181b-5p	AACAUUCAUUGCUGUCGGUGGG	L	−1.75554
miR-107a-3p	AGCAGCAUUGUACAGGGCUAUCA	L	−2.81985
miR-101a	UACAGUACUGUGAUAACUGAAG	L	−3.05599
miR-181c-3p	CUCGCCGGACAAUGAAUGAGAA	L	−5.01275
miR-103	AGCAGCAUUGUACAGGGCUAUGA	L	−2.61915
miR-146b	UGAGAACUGAAUUCCAAGGGUG	L	−3.91539
miR-138-5p	AGCUGGUGUUGUGAAUCAGGCC	L	−4.94236
miR-429a	UAAUACUGUCUGGUAAUGCCGU	L	−2.44487
miR-153b-5p	GUCAUUUUUGUGGUUUGCAGCU	L	−3.67933
miR-125c-5p	UCCCUGAGACCCUAACUCGUGA	L	−5.07926
miR-181a-5p	AACAUUCAACGCUGUCGGUGAGU	L	−1.97076
miR-199-3-3p	ACAGUAGUCCGCACAUUGGUU	L	−3.81683
miR-125b-5p	UCCCUGAGACCCUAACUUGUGA	L	−3.43276
miR-26a-5p	UUCAAGUAAUCCAGGAUAGGCU	L	−3.35436
miR-722	UUUUUUGCAGAAACGUUUCAGAUU	L	−6.25146
miR-222a-3p	AGCUACAUCUGGCUACUGGGUCUC	L	−3.55456

* I24: SSN-1 cells infected with SHVV (24 h); C24: SSN-1 cells (24 h); N: nucleoprotein; P: phosphoprotein; M: matrix protein; G: glycoprotein; L: RNA-dependent RNA polymerase protein (large protein).

### 2.6. Effects of Three Selected miRNAs on SHVV Multiplication

**Figure 5:**
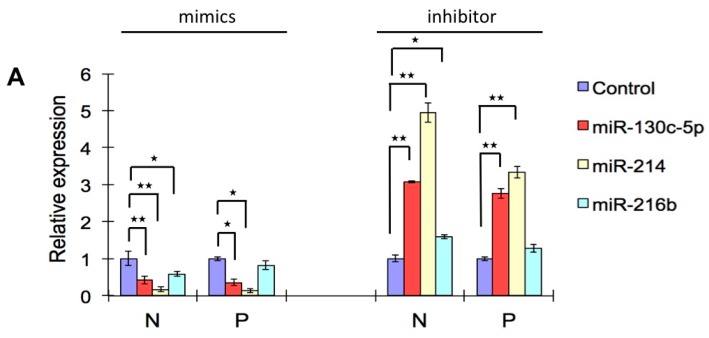

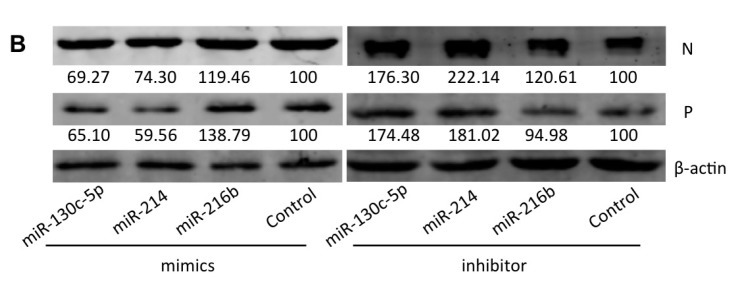
Effects of three differentially-expressed miRNAs on SHVV multiplication. (**A**) Quantification of mRNA level of N and P genes of SHVV via qRT-PCR. The mimics or inhibitors of three selected miRNAs, as well as the mimic control or the inhibitor control were transfected into SSN-1 cells followed by SHVV infection. * and ** respectively indicate statistically-significant differences between infected and non-infected samples (* *p* < 0.05; ** *p* < 0.01); (**B**) Detection of protein level of N and P genes of SHVV via Western blotting. β-actin was used as the internal control protein. The integrated optical densities of the protein bands were measured using Image-Pro Plus 6.0. The value of the N and P protein bands are normalized to β-actin. The value of the N and P protein bands of the control were set as 100, while the values of the N and P protein bands of the three miRNAs were compared to that of the control.

Three differentially-expressed miRNAs, including miR-130-5p, miR-214 and miR-216b, were chosen to evaluate their effects on SHVV multiplication. miR-130-5p and miR-216b were predicted to bind to N, G and L genes, while miR-214 was predicted to bind to N and P genes (Table 2). The mimics and inhibitors of the three miRNAs were used to treat SSN-1 cells followed by SHVV infection. The results of qRT-PCR showed that all three miRNAs exhibited significant inhibitory effects on mRNA expression of the N gene, while for the P gene, only miR-130c-5p and miR-214 exhibited inhibitory effects (Figure 5A). Among the three miRNAs, miR-214 exhibited the most significant inhibitory effect (Figure 5A). Western blotting was also performed to evaluate the effects of these three miRNAs on protein expression of N and P genes. Similar to the effects on mRNA expression of SHVV, miR-130c-5p and miR-214 also exhibited a significant inhibitory effect on protein expression of SHVV. Together, these data demonstrated the three miRNAs, at least miR-130c-5p and miR-214, could efficiently repress SHVV multiplication.

## 3. Discussion

Although miRNAs are widely expressed in a variety of organisms, their expression profiles vary greatly among tissues [21]. For example, miR-122 is highly expressed in hepatocytes, but is absent in almost all other cells [22]. Therefore, different miRNA expression profiles might be acquired when we use different tissues from the same organism. The miRNA expression profile of several teleost fishes has recently been identified in different tissues, including bighead carp and silver carp [23], common carp [24], blunt snout bream [25] and epithelial cells of carp [14]. Since SHVV could infect and replicate in several tissues of snakehead fish [18], we choose SNN-1 cells, which are derived from a whole snakehead fry fish [26], to study the differential miRNA expression profile of snakehead fish upon SHVV infection.

Upon infection, viruses encounter a large number of miRNAs expressed in host cells. Differentially-expressed miRNAs pre- and post-virus infection may be associated with viral infection [27]. In this study, we identified 143 known and seven novel differentially-expressed miRNAs in SSN-1 cells upon SHVV infection at 24 h poi. Interestingly, all of the 143 known differentially-expressed miRNAs were downregulated (Table S3). However, two of the seven novel miRNAs were upregulated and five were downregulated (Table 1), and the reasons are not known. Functional confirmation of the 150 differentially-expressed miRNAs in SSN-1 cells to facilitate or repress virus multiplication is needed.

Multiple reports have revealed that miRNAs regulate virus replication by targeting key elements of innate immunity. miR-136 regulates host antiviral innate immunity by acting as an immune agonist of retinoic acid-inducible gene 1 (RIG-1) [28]. miR-146 was identified as a regulator of enterovirus replication by targeting key elements of the MyD88 signal pathway [29]. In addition, miRNAs also mediate antiviral defense by targeting viral transcripts. miR-122 stimulates translation of hepatitis C virus RNA through interaction with the 3′ UTRs of viral genome [30]. miR-181 can directly impair porcine reproductive and respiratory syndrome virus replication via specifically binding to a conserved region in the downstream of open reading frame 4 of the viral genomic RNA [31]. miR-let-7c inhibits H1N1 influenza virus replication by targeting the 3′ UTR of the M1 genome and represses M1 protein expression [32]. In this study, three downregulated miRNAs that were predicted to target two or three genes of SHVV were selected to determine their effects on virus multiplication. All three miRNAs exhibited significant antiviral properties. In addition to binding to the SHVV genome, these three miRNAs were predicted to target tens of host genes (data not shown). Some of the predicted host genes are involved in antiviral signal pathways. Therefore, it is possible that these miRNAs utilize both direct and indirect ways to suppress SHVV multiplication. Further studies are needed to understand the exact mechanisms of the inhibition.

The miRNA was not exclusively host miRNA, many viruses can encode viral miRNAs, which not only facilitate viral replication, but also help the virus to counteract host immune response. Based on the ability of viral miRNA production, viruses can be divided into three categories: the herpesviruses, which encode multiple viral miRNAs; the nuclear DNA viruses, which may encode one or two miRNAs; the RNA viruses and cytoplasmic DNA viruses, which appear to lack any miRNAs [7]. In this study, no miRNA encoded by SHVV was identified. The probable reason behind this seems to be that SHVV is an RNA virus.

## 4. Experimental Section

### 4.1. Virus, Cells and RNA Preparation

SHVV was isolated from diseased hybrid snakehead fish and stored at −80 °C. SSN-1 cells, kindly provided by Hong Liu from Shenzhen Animal & Plant Inspection and Quarantine Technology Center, were cultured in minimum essential medium (HyClone, Logan, UT, USA) supplemented with 10% fetal bovine serum (Gibco, Auckland, New Zealand) and penicillin (100 µg/mL), streptomycin (100 µg/mL) at 25 °C. To prepare RNA sample, SSN-1 cells were grown on four 6-well plastic dishes (Costar, Corning, NY, USA). Two 6-well plastic dishes was infected with 0.1 multiplicity of infection (MOI) of SHVV; the other two were used as uninfected controls with an equal volume of PBS. At 3 and 24 h poi, SHVV infected and non-infected SSN-1 cells were collected in TRIzol reagent (Invitrogen, Carlsbad, CA, USA) for the total RNA extraction according to the manufacturer’s instructions.

### 4.2. Small RNA Library Construction 

The RNA samples were subjected to 15% polyacrylamide gel electrophoresis (PAGE) for the enrichment of molecules with a length of 17–30 nt [14]. After 5′ and 3′ adaptors were added, RT-PCR with adaptor-specific primers was used to generate a small RNA library from SHVV-infected and non-infected SSN-1 cells at 3 and 24 h poi. The small RNA library was then sequenced by illumine Hiseq2500.

### 4.3. Sequencing Data Analysis

The raw reads were counted and the clean reads of four samples, including SSN-1 cells (3 h), SSN-1 cells infected with SHVV (3 h), SSN-1 cells (24 h) and SSN-1 cells infected with SHVV (24 h), were acquired by excluding low-quality reads, 3′ adapter reads, 5′ adapter reads and poly(A) sequences. The length distribution of the clean reads was calculated, and the clean reads were aligned with the genome of zebra fish. Conserved miRNAs were identified by aligning the small RNAs of the four samples to the Zebrafish miRNAs data in miRBase Version 20.0 [33].

### 4.4. Identification of Novel miRNAs

MiRDeep2 software was used to identify potential novel miRNAs from the four samples. Overlapping sequences were used to form longer sequences according to their alignments to known precursor sequences in the miRBase. The differentially-expressed novel miRNAs were summarized between SHVV-infected and non-infected SSN-1 cells.

### 4.5. Analysis of Differentially-Expressed miRNAs

The identified miRNAs in the four samples were analyzed to identify differentially-expressed miRNAs between SHVV-infected and non-infected SSN-1 cells. The fold change higher than 2 and a *p*-value <0.05 were the criteria to select differentially-expressed miRNAs.

### 4.6. Prediction of miRNA Targets on the SHVV Genome

The miRNA targets on the SHVV genome were predicted using Miranda software [34].

### 4.7. RT-PCR and qRT-PCR Analysis of miRNAs

The predicted novel miRNAs were validated by RT-PCR as described previously [14]. Five randomly-selected miRNAs were detected by qRT-PCR using the same RNA samples used for the construction of the miRNA library. Five forward primers were designed based on mature miRNA sequences (Table S5). A 20-bp segment at the 3′ end of the 5S rRNA gene was amplified as an endogenous control to normalize template amounts. Quantitative PCR reactions were conducted in 20-µL volumes containing 1 µL diluted cDNA, 300 nM of each primer and 10 µL of the SYBR Master Mix with the following cycling conditions: 95 °C for 5 min, 45 cycles at 95 °C for 10 s, 58 °C for 10 s and 72 °C for 15 s, and ended with a 95 °C at 5 °C/s calefactive velocity to make the melt curve. All expression levels were normalized to the arithmetic mean of the selected 5S ribosomal RNA gene. Amplification results were analyzed using a comparative *C*_t_ method, which uses a formula 2^−ΔΔ*C*t^ to achieve results for relative quantification. *C*_t_ represents the threshold cycle.

### 4.8. Effects of Three Randomly-Selected Differentially-Expressed miRNAs on Virus Multiplication

SSN-1 cells in 12-well plates were transfected with 100 nM mimics or 50 nM inhibitors of miR-130c-5p, miR-214, miR-216b, as well as mimic control or inhibitor control using Lipofectamine 2000 (Invitrogen, USA). The inhibitor is the single-strand RNA that is completely paired to miRNAs. At 24 h post-transfection, the cells were infected with 0.1 MOI of SHVV for 24 h. The cells were harvested to determine the effects of three miRNA mimics or inhibitors on virus mRNA expression by qRT-PCR and protein expression by Western blotting.

## Supplementary Materials

Supplementary materials can be found at http://www.mdpi.com/1422-0067/17/2/154/s1.

## References

[1] Fan H.X., Tang H. (2014). Complex interactions between microRNAs and hepatitis B/C viruses. World J. Gastroenterol..

[2] Wang X.G., Yu J.F., Zhang Y., Gong D.Q., Gu Z.L. (2012). Identification and characterization of microRNA from chicken adipose tissue and skeletal muscle. Poult. Sci..

[3] Djuranovic S., Nahvi A., Green R. (2012). miRNA-mediated gene silencing by translational repression followed by mRNA deadenylation and decay. Science.

[4] Trakooljul N., Hicks J.A., Liu H.C. (2010). Identification of target genes and pathways associated with chicken microRNA miR-143. Anim. Genet..

[5] Bartel D.P. (2009). MicroRNAs, target recognition and regulatory functions. Cell.

[6] Doench J.G., Sharp P.A. (2004). Specificity of microRNA target selection in translational repression. Genes Dev..

[7] Cullen B.R. (2010). Five questions about viruses and microRNAs. PLoS Pathog..

[8] Skalsky R.L., Cullen B.R. (2010). Viruses, microRNAs, and host interactions. Annu. Rev. Microbiol..

[9] Cullen B.R. (2011). Viruses and microRNAs, RISCy interactions with serious consequences. Genes Dev..

[10] Xiao C., Calado D.P., Galler G., Thai T.H., Patterson H.C., Wang J., Rajewsky N., Bender T.P., Rajewsky K. (2007). MiR-150 controls B cell differentiation by targeting the transcription factor c-Myb. Cell.

[11] Xu P., Vernooy S.Y., Guo M., Hay B.A. (2003). The Drosophila microRNA Mir-14 suppresses cell death and is required for normal fat metabolism. Curr. Biol..

[12] Ou J., Meng Q., Li Y., Xiu Y., Du J., Gu W., Wu T., Li W., Ding Z., Wang W. (2012). Identification and comparative analysis of the Eriocheir sinensis microRNA transcriptome response to Spiroplasma eriocheiris infection using a deep sequencing approach. Fish Shellfish Immunol..

[13] Trobaugh D.W., Gardner C.L., Sun C., Haddow A.D., Wang E., Chapnik E., Mildner A., Weaver S.C., Ryman K.D., Klimstra W.B. (2014). RNA viruses can hijack vertebrate microRNAs to suppress innate immunity. Nature.

[14] Wu S., Liu L., Zohaib A., Lin L., Yuan J., Wang M., Liu X. (2015). MicroRNA profile analysis of Epithelioma papulosum cyprini cell line before and after SVCV infection. Dev. Comp. Immunol..

[15] Betts A.M., Stone D.M., Way K., Torhy C., Chilmonczyk S., Benmansour A., de Kinkelin P. (2003). Emerging vesiculo-type virus infections of freshwater fishes in Europe. Dis. Aquat. Organ..

[16] Crane M., Hyatt A. (2011). Viruses of fish, an overview of significant pathogens. Viruses.

[17] McFee R.B. (2007). Global infections—Avian influenza and other significant emerging pathogens: An overview. Dis. Mon..

[18] Liu X., Wen Y., Hu X., Wang W., Liang X., Li J., Vakharia V., Lin L. (2015). Breaking the host range: Mandarin fish is susceptible to a vesiculovirus derived from snakehead fish. J. Gen. Virol..

[19] Zeng W., Wang Q., Wang Y., Liu C., Liang H., Fang X., Wu S. (2014). Genomic characterization and taxonomic position of a rhabdovirus from a hybrid snakehead. Arch. Virol..

[20] Wang W., Asim M., Yi L., Hegazy A.M., Hu X., Zhou Y., Ai T., Lin L. (2015). Abortive infection of snakehead fish vesiculovirus in ZF4 cells was associated with the RLRs pathway activation by viral replicative intermediates. Int. J. Mol. Sci..

[21] Landgraf P., Rusu M., Sheridan R., Sewer A., Iovino N., Aravin A., Pfeffer S., Rice A., Kamphorst A.O., Landthaler M. (2007). A mammalian microRNA expression atlas based on small RNA library sequencing. Cell.

[22] Cullen B.R. (2013). How do viruses avoid inhibition by endogenous cellular microRNAs?. PLoS Pathog..

[23] Chi W., Tong C., Gan X., He S. (2011). Characterization and comparative profiling of MiRNA transcriptomes in bighead carp and silver carp. PLoS ONE.

[24] Yan X., Ding L., Li Y., Zhang X., Liang Y., Sun X., Teng C.B. (2012). Identification and profiling of microRNAs from skeletal muscle of the common carp. PLoS ONE.

[25] Yi S., Gao Z.X., Zhao H., Zeng C., Luo W., Chen B., Wang W.M. (2013). Identification and characterization of microRNAs involved in growth of blunt snout bream (*Megalobrama amblycephala*) by Solexa sequencing. BMC Genom..

[26] Frerichs G.N., Morgan D., Hart D., Skerrow C., Roberts R.J., Onions D.E. (1991). Spontaneously productive C-type retrovirus infection of fish cell lines. J. Gen. Virol..

[27] Li Z.J., Zhang Y.P., Li Y., Zheng H.W., Zheng Y.S., Liu C.J. (2014). Distinct expression pattern of miRNAs in Marek’s disease virus infected-chicken splenic tumors and non-tumorous spleen tissues. Res. Vet. Sci..

[28] Zhao L., Zhu J., Zhou H., Zhao Z., Zou Z., Liu X., Lin X., Zhang X., Deng X., Wang R. (2015). Identification of cellular microRNA-136 as a dual regulator of RIG-I-mediated innate immunity that antagonizes H5N1 IAV replication in A549 cells. Sci. Rep..

[29] Taganov K.D., Boldin M.P., Chang K.J., Baltimore D. (2006). NF-ĸB-dependent induction of microRNA miR-146, an inhibitor targeted to signaling proteins of innate immune responses. Proc. Natl. Acad. Sci. USA.

[30] Henke J.I., Goergen D., Zheng J., Song Y., Schuttler C.G., Fehr C., Junemann C., Niepmann M. (2008). microRNA-122 stimulates translation of hepatitis C virus RNA. EMBO J..

[31] Guo X.K., Zhang Q., Gao L., Li N., Chen X.X., Feng W.H. (2013). Increasing expression of microRNA 181 inhibits porcine reproductive and respiratory syndrome virus replication and has implications for controlling virus infection. J. Virol..

[32] Ma Y.J., Yang J., Fan X.L., Zhao H.B., Hu W., Li Z.P., Yu G.C., Ding X.R., Wang J.Z., Bo X.C. (2012). Cellular microRNA let-7c inhibits M1 protein expression of the H1N1 influenza A virus in infected human lung epithelial cells. J. Cell. Mol. Med..

[33] miRBase. http://www.mirbase.org.

[34] Miranda. http://www.microrna.org/microrna/home.do.

