# Supplementary material for: Identification and Characterization of MicroRNAs in Snakehead Fish Cell Line upon Snakehead Fish Vesiculovirus Infection
Source: Int J Mol Sci. 2016 Jan 26;17(2):154. doi: 10.3390/ijms17020154 (PMC4783888; doi:10.3390/ijms17020154)
Supplement: Supplementary file 1 [file ijms-17-00154-s001.zip › ijms-115486-Supplementary Materials/ijms-115486-Supplementary.pdf]

Supplementary Materials: Identification and Characterization of MicroRNAs in Snakehead Fish Cell Line upon Snakehead Fish Vesiculovirus Infection

Xiaodan Liu, Jiagang Tu, Junfa Yuan, Xueqin Liu, Lijuan Zhao, Farman Ullah Dawar, Muhammad Nasir Khan Khattak, Abeer M. Hegazy, Nan Chen, Vikram N. Vakharia and Li Lin

Table S1. Statistics of the preliminary sequencing of SSN-1 cells with or without SHVV infection.

Sample	Clean Reads	Unique Clean Reads	Aligned * Unique Clean Reads (%)
SSN-1 cell (3 h)	11,049,428	396,669	151,939 (38.30%)
SSN-1 cell infected with SHVV (3 h)	9,754,366	280,855	88,889 (31.64%)
SSN-1 cell (24 h)	13,125,705	340,204	131,607 (38.68%)
SSN-1 cell infected with SHVV (24 h)	16,815,529	1,022,296	210,524 (20.59%)

* The unique clean reads were aligned with the genome of *Danio rerio*.

Table S2. Differentially expressed miRNAs ($p < 0.05$) when the sample SSN-1 cell infected with SHVV (3 h) was compared to SSN-1 cell (3 h).

Name	Fold Change ($Log2^{I3/C3}$)*	*p* Value
miR-126a-5p>miR-126b-5p	1.13966	2.90×10^{-10}
miR-135c	2.50178	0.000294
miR-140-5p	1.1076	3.47×10^{-17}
miR-143	1.18943	1.70×10^{-9}
miR-145-5p	1.91682	0.032896
miR-146a	1.61184	0
miR-153a-3p	1.25586	1.30×10^{-38}
miR-153c-3p	1.09739	1.32×10^{-8}
miR-183-5p	1.51688	4.54×10^{-5}
miR-184	3.17985	0.012661
miR-7147	1.10041	0.000831
miR-455-5p	1.36427	0.006879
miR-199-5p	1.20884	0
miR-221-5p	1.25024	0.020809
miR-24	1.03277	7.36×10^{-51}
miR-31	−1.97543	1.30×10^{-15}
miR-196d	−4.06808	6.62×10^{-5}
miR-203a-3p	−9.77787	2.35×10^{-27}

* I3: SSN-1 cell infected with SHVV (3 h); C3: SSN-1 cell (3 h).

Table S3. Differentially expressed miRNAs ($p < 0.05$) when the sample SSN-1 cell infected with SHVV (24 h) was compared to SSN-1 cell (24 h).

Name	Fold Change ($Log_2{}^{I24/C24}$)*	*p* Value
let-7a	−3.23603	0
let-7b	−3.14206	0
let-7c-3p>let-7d-3p	−4.00125	1.23×10^{-7}
let-7c-5p	−2.58041	3.58×10^{-133}
let-7d-5p	−2.99895	2.66×10^{-32}
let-7e	−3.35087	0

Table S3. *Cont.*

Name	Fold Change ($Log_2{}^{I24/C24}$)*	p Value
let-7f	−3.29203	0
let-7g	−2.732	0
let-7h	−2.51911	0
let-7i	−2.29029	9.44×10^{-99}
let-7j	−3.0519	0
miR-100-3p	−3.61793	1.18×10^{-163}
miR-100-5p	−5.84746	0
miR-101a	−3.05599	6.30×10^{-237}
miR-101b	−2.73747	0
miR-103	−2.61915	8.57×10^{-304}
miR-107a-3p	−2.81985	1.02×10^{-221}
miR-10a-3p	−4.60532	5.79×10^{-12}
miR-10a-5p	−2.1018	0
miR-10b-2-3p	−3.94807	1.89×10^{-65}
miR-10b-5p	−2.39311	0
miR-10d-5p	−2.63848	0
miR-125a	−3.16476	1.38×10^{-33}
miR-125b-2-3p	−5.51039	0
miR-125b-5p	−3.43276	0
miR-125c-5p	−5.07926	4.37×10^{-284}
miR-126a-3p	−4.2054	1.78×10^{-20}
miR-126a-5p>miR-126b-5p	−2.66274	1.47×10^{-31}
miR-128-3p	−4.92859	0
miR-1306	−3.21539	1.84×10^{-7}
miR-130a	−3.11229	0
miR-130c-3p	−3.38263	0
miR-130c-5p	−4.79246	6.23×10^{-120}
miR-132-3p	−3.89345	1.75×10^{-30}
miR-135c	−1.6469	0.002498916
miR-138-5p	−4.94236	1.53×10^{-29}
miR-1388-3p	−2.04562	2.08×10^{-126}
miR-140-3p	−3.03951	0
miR-140-5p	−3.46115	2.52×10^{-112}
miR-143	−3.5958	7.15×10^{-37}
miR-145-3p	−6.05784	6.42×10^{-18}
miR-145-5p	−7.017	7.15×10^{-7}
miR-146a	−1.98658	0
miR-146b	−3.91539	9.52×10^{-55}
miR-148	−3.23654	6.54×10^{-111}
miR-150	−2.63751	3.77×10^{-13}
miR-153a-3p	−2.95278	6.56×10^{-163}
miR-153b-3p	−2.41897	3.60×10^{-57}
miR-153b-5p	−3.67933	6.62×10^{-11}
miR-153c-3p	−2.27796	1.80×10^{-24}
miR-15a-5p	−4.25559	0
miR-15b-5p	−3.87608	0
miR-16a	−4.02428	0
miR-16b	−2.99958	0

Table S3. *Cont.*

Name	Fold Change (Log_2 $^{I24/C24}$)*	*p* Value
miR-16c-5p	−1.91909	0
miR-17a-2-3p	−3.23186	5.89×10^{-6}
miR-17a-3p	−3.16475	1.29×10^{-11}
miR-17a-5p	−3.15537	0
miR-181a-3p	−3.1716	0
miR-181a-5p	−1.97076	0
miR-181b-5p	−1.75554	0
miR-181c-3p	−5.01275	1.17×10^{-38}
miR-181c-5p	−1.84696	3.98×10^{-70}
miR-183-5p	−1.57979	0.000204525
miR-184	−2.16476	0.004007859
miR-18a	−3.26618	1.23×10^{-83}
miR-192	−4.12293	1.60×10^{-72}
miR-193a-3p	−4.76167	0
miR-196a-5p	−2.96858	0
miR-199-3-3p	−3.81683	1.38×10^{-74}
miR-199-3p	−2.97786	0
miR-199-5p	−3.96545	0
miR-19a-3p	−1.85964	0
miR-19b-3p	−2.03149	0
miR-19b-5p	−7.73689	8.22×10^{-11}
miR-19c-3p	−3.0051	3.09×10^{-57}
miR-19d-3p	−2.12699	0
miR-204-5p	−3.14817	1.68×10^{-144}
miR-205-5p	−3.74971	2.46×10^{-6}
miR-20a-5p	−3.19422	0
miR-21	−3.39804	0
miR-210-3p	−3.67693	0
miR-210-5p	−5.51449	0.006223011
miR-212	−2.62749	1.63×10^{-15}
miR-214	−4.27387	8.54×10^{-123}
miR-216b	−6.73689	8.49×10^{-6}
miR-221-3p	−2.48955	0
miR-221-5p	−5.56686	1.10×10^{-12}
miR-222a-3p	−3.55456	0
miR-222a-5p	−2.8953	0
miR-22a-3p	−3.09154	0
miR-22a-5p	−3.04137	7.28×10^{-80}
miR-22b-3p	−4.02414	0
miR-22b-5p	−2.84283	1.22×10^{-27}
miR-23a-3-5p	−7.92953	3.04×10^{-12}
miR-23a-3p	−2.13028	0
miR-23a-5p	−6.09946	0.000524275
miR-23b	−2.73405	4.47×10^{-250}
miR-24	−3.40993	0
miR-25-3p	−2.31239	0
miR-26a-2-3p	−3.8599	9.31×10^{-10}
miR-26a-5p	−3.35436	0

Table S3. *Cont.*

Name	Fold Change (Log2 $^{I24/C24}$)*	*p* Value
miR-26b	−3.6547	0
miR-27a-3p	−2.4537	0
miR-27b-3p	−3.29876	0
miR-27b-5p	−3.24885	1.49×10^{-189}
miR-27c-3p	−2.77851	0
miR-27d	−3.95566	4.61×10^{-29}
miR-27e	−4.40179	2.76×10^{-10}
miR-29a	−3.25812	0
miR-29b	−3.20471	1.75×10^{-178}
miR-301a	−2.1425	2.71×10^{-27}
miR-301b-3p	−4.92954	0.032381332
miR-301c-3p	−2.45693	4.21×10^{-6}
miR-30b	−3.714	0
miR-30c-5p	−2.58964	3.93×10^{-59}
miR-30d	−3.15037	0
miR-30e-3p	−3.06374	0
miR-30e-5p	−3.38238	0
miR-31	−2.45693	4.21×10^{-6}
miR-338	−4.16476	8.00×10^{-5}
miR-34a	−2.5756	0
miR-363-3p	−3.26429	0.000196783
miR-365	−4.92954	0.032381332
miR-375	−3.3029	0
miR-429a	−2.44487	0.000616735
miR-454b	−2.98676	5.94×10^{-21}
miR-455-5p	−2.3574	0.000266601
miR-456	−3.03714	1.36×10^{-70}
miR-457a	−3.01561	1.53×10^{-21}
miR-462	−2.53661	7.36×10^{-231}
miR-499-3p	−5.25146	0.014195401
miR-7147	−4.7948	4.43×10^{-20}
miR-722	−6.25146	0.000229833
miR-724	−2.63081	2.42×10^{-106}
miR-730	−4.60532	5.79×10^{-12}
miR-731	−3.18425	3.20×10^{-188}
miR-737-5p	−3.65232	0
miR-9-4-3p	−4.65508	4.74×10^{-18}
miR-9-5p	−1.54182	0.002096158
miR-92a-3p	−3.97976	0
miR-92b-3p	−4.75081	0
miR-99	−4.71935	4.02×10^{-283}

* I24: SSN-1 cell infected with SHVV (24 h); C24: SSN-1 cell (24 h).

Table S4. Summary of targeted virus gene prediction of the differentially expressed miRNAs when the sample SSN-1 cell infected with SHVV (3 h) was compared to SSN-1 cell (3 h).

Name	Sequence (5′→3′)	Target	Fold Change ($Log_2{}^{I3/C3}$)*
miR-199-5p	CCCAGUGUUCAGACUACCUGUUC	N, L	1.20884
miR-135c	UAUGGCUUUCUAUUCCUAUGUG	M	2.50178
miR-7147	UGUACCAUGCUGGUAGCCAGU	G	1.10041
miR-145-5p	GUCCAGUUUUCCCAGGAAUCCC	G	1.91682
miR-184	UGGACGGAGAACUGAUAAGGGC	G	3.17985
miR-153a-3p	UUGCAUAGUCACAAAAGUGAUC	G, L	1.25586
miR-146a	UGAGAACUGAAUUCCAUAGAUGG	L	1.61184
miR-153c-3p	UUGCAUAGUCACAAAAAUGAUC	L	1.09739
miR-183-5p	UAUGGCACUGGUAGAAUUCACUG	L	1.51688
miR-203a-3p	GUGAAAUGUUUAGGACCACUUG	L	−9.77787

* I3: SSN-1 cell infected with SHVV (3 h), C3: SSN-1 cell (3 h). N: nucleoprotein; P: phosphoprotein; M: matrix protein; G: glycoprotein; L: RNA-dependent RNA polymerase protein (large protein).

Table S5. Primers sequences for RT-PCR and qRT-PCR.

Name	RT Primers	qRT-PCR	qRT-PCR
	(5′-3′)	Forward primers (5′-3′)	Reverse primers (5′-3′)
miR-135c	Unique Oligo-dT Adaptor primer *	TATGGCTTTCTATTCCTATGTG	Universal primer *
miR-140-5p	As above	CAGTGGTTTTACCCTATGGTAG	As above
miR-153a-3p	As above	TTGCATAGTCACAAAAGTGATC	As above
miR-153c-3p	As above	TTGCATAGTCACAAAAATGATC	As above
miR-24	As above	TGGCTCAGTTCAGCAGGAACAG	As above
5S rRNA	Oligo-dT primer and Random 6	GGAGACCGCCTGGGAATA	As above
N	As above	CCGCATCGGAAATCAAGCAG	GTTGACCGCTTGCCCAATTT
P	As above	ACAGCTATCCTCAAGCCGTG	ACAGCACCATTTGCTGAACC
β-actin	As above	CACTGTGCCCATCTACGAG	CCATCTCCTGCTCGAAGTC

*: provided from All-in-One™ miRNA qRT-PCR Detection Kit.
